# Evolution of Complex RNA Polymerases: The Complete Archaeal RNA Polymerase Structure

**DOI:** 10.1371/journal.pbio.1000102

**Published:** 2009-05-05

**Authors:** Yakov Korkhin, Ulug M Unligil, Otis Littlefield, Pamlea J Nelson, David I Stuart, Paul B Sigler, Stephen D Bell, Nicola G. A Abrescia

**Affiliations:** 1 Department of Molecular Biophysics and Biochemistry, Yale University, New Haven, Connecticut, United States of America; 2 Howard Hughes Medical Institute, Yale University, New Haven, Connecticut, United States of America; 3 Harvard Medical School, Boston, Massachusetts, United States of America; 4 Howard Hughes Medical Institute, Harvard University, Boston, Massachusetts, United States of America; 5 Division of Structural Biology and the Oxford Protein Production Facility, The Wellcome Trust Centre for Human Genetics, University of Oxford, Oxford, United Kingdom; 6 Sir William Dunn School of Pathology, University of Oxford, Oxford, United Kingdom; 7 Structural Biology Unit, CIC bioGUNE, Derio, Spain; Vanderbilt University, United States of America

## Abstract

The archaeal RNA polymerase (RNAP) shares structural similarities with eukaryotic RNAP II but requires a reduced subset of general transcription factors for promoter-dependent initiation. To deepen our knowledge of cellular transcription, we have determined the structure of the 13-subunit DNA-directed RNAP from Sulfolobus shibatae at 3.35 Å resolution. The structure contains the full complement of subunits, including RpoG/Rpb8 and the equivalent of the clamp-head and jaw domains of the eukaryotic Rpb1. Furthermore, we have identified subunit Rpo13, an RNAP component in the order Sulfolobales, which contains a helix-turn-helix motif that interacts with the RpoH/Rpb5 and RpoA′/Rpb1 subunits. Its location and topology suggest a role in the formation of the transcription bubble.

## Author Summary

Transcription, the process of converting DNA into RNA (which in turn is translated into proteins by ribosomes) is carried out by the multisubunit RNA polymerase (RNAP) enzyme. Transcription is fundamental to all organisms across the three kingdoms of life—Eukarya, Bacteria, and Archaea—and can be divided into three major steps: initiation, transcription/elongation, and termination. Eukaryotes have three different nuclear RNAPs, whereas Archaea and Bacteria have one. Archaeal transcription is similar to that of eukaryotes, but initiation requires only two accessory proteins bound to DNA: transcription factor B (TFB) and TATA-box binding protein (TBP). It is believed that studies of the archaeal enzyme may shed light on the more complex eukaryotic RNAP. Our complete structure of the archaeal RNAP from Sulfolobus shibatae has fully elucidated its architecture, confirming its close evolutionary relationship with the eukaryotic RNAP II and at the same time revealed a new subunit, Rpo13, with no ortholog in the eukaryotic enzyme. The location and topology of Rpo13 allow us to suggest a mechanism by which Archaea bypass the additional eukaryotic cofactors required for transcription initiation.

## Abstract 3

The complete archaeal RNA polymerase structure is strikingly similar to the equivalent eukaryotic enzyme. Rpo13, a newly identified archaeal-specific subunit, may explain the minimal set of cofactors required for archaeal transcription initiation.

## Introduction

Gene expression in cellular organisms across the three kingdoms of life is carried out by multisubunit RNA polymerase (RNAP) enzymes. Eukaryotes have three different multisubunit nuclear RNAPs (Pol I, II, and III), whereas Archaea and Bacteria have single enzymes [1]. A wealth of structural information has been gathered in the past decade allowing the visualization of RNAP II in isolation, in the act of transcription, and in complex with transcription factors transcription factor II S (TFIIS) or transcription factor II B (TFIIB) [2–5]. The archaeal transcription machinery is orthologous to that of eukaryotes, but initiation only requires two accessory factors: transcription factor B (TFB) (an ortholog of TFIIB) and TATA-box binding protein (TBP) [6–8], and thus provides a simplified model system for studying transcription initiation. In eukaryotes, the additional basal factors are needed, in part to facilitate DNA melting at the initiation site, a functional complexity that Archaea must overcome by other means [6,7]. Recent structural studies on archaeal polymerases [9,10] have shed light on the basic architecture, but the information gathered thus far remains incomplete.

**Figure 1:**
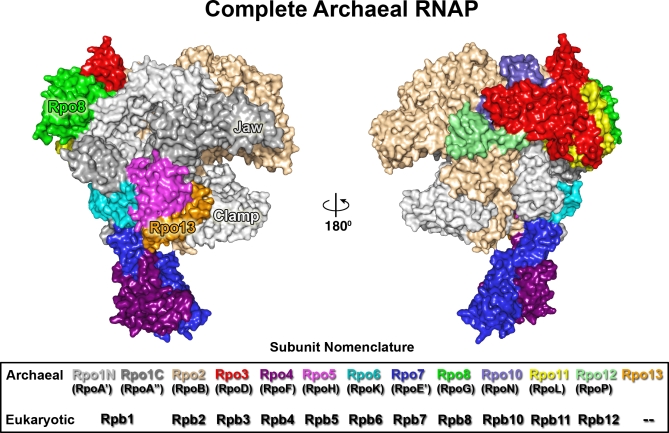
Surface Representation of the Complete Archaeal RNA Polymerase from S. shibatae The overall architecture of the RNAP is shown with the different subunits colour-coded as illustrated in the Subunit Nomenclature (below). The newly located structural elements are labelled.

We have therefore determined the crystal structure of the complete DNA-directed RNA polymerase from the archaeon Sulfolobus shibatae (SshRNAP) at 3.35 Å resolution (see Materials and Methods and Table 1) revealing the complete 13 subunit set of the functional enzyme including two subunits so far undetected: RpoG/Rpb8 and Rpo13 (Figure 1). The highly positively charged C-terminus of Rpo13 extends into the DNA entry channel, suggesting its involvement in binding to nucleic acids. Our intact RNAP structure allows us to propose a model for the archaeal preinitiation complex formation.

**Table 1:**
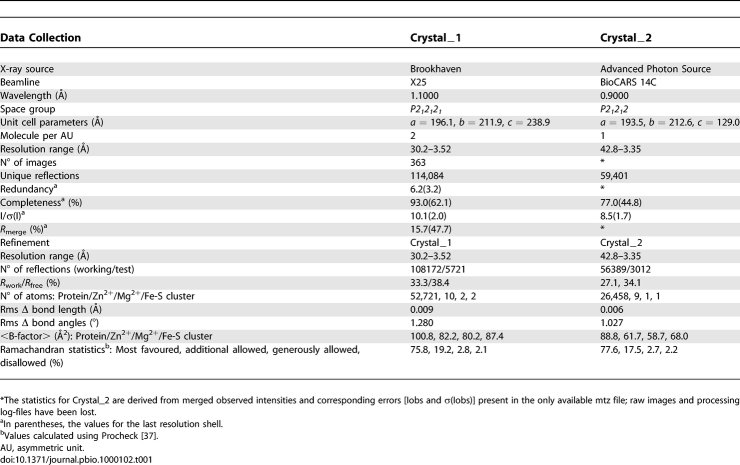
Data Collection and Refinement Statistics

## Results

### Overall RNA Polymerase Structure

**Figure 2:**
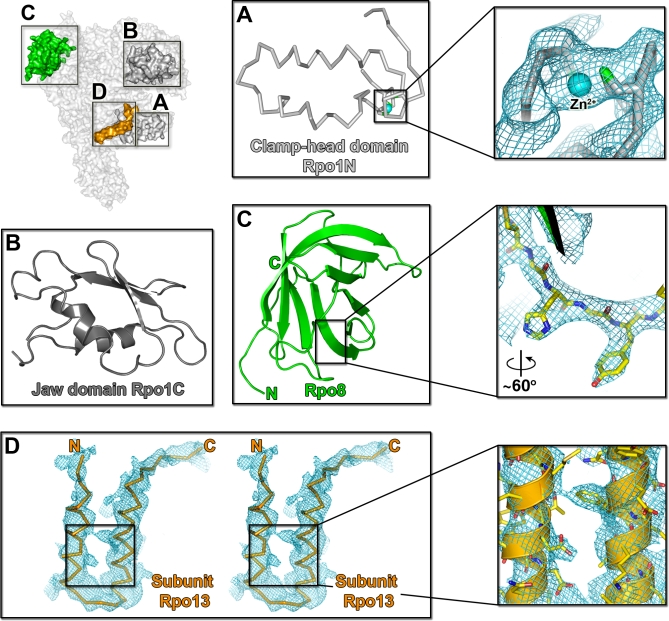
Newly Identified Subunits and Located Subdomains (A) Ribbon representation of the clamp-head domain in Rpo1N (light grey); C98, C101, and C146 as green stick; and Zn ion as a cyan sphere. On the right, sigmaA-weighted 2Fo-Fc electron density countered at 1σ (as a blue mesh) corresponding to the Zn^2+^ and surrounding cysteines (arbitrary view). (B) Cartoon representation of the jaw domain in the Rpo1C subunit. (C) Cartoon representation of the entire Rpo8 subunit. On the right, sigmaA-weighted 2Fo-Fc electron density countered at 1.2σ of a structural detail. (D) Stereo view of the sigmaA-weighted 2Fo-Fc map electron density (contoured at 1.1σ) correspondent to the HTH motif of the Rpo13 subunit (as orange ribbon with N and C labelling the N- and C-termini). The inset shows some side chains (as sticks) fitting density.

**Figure 3:**
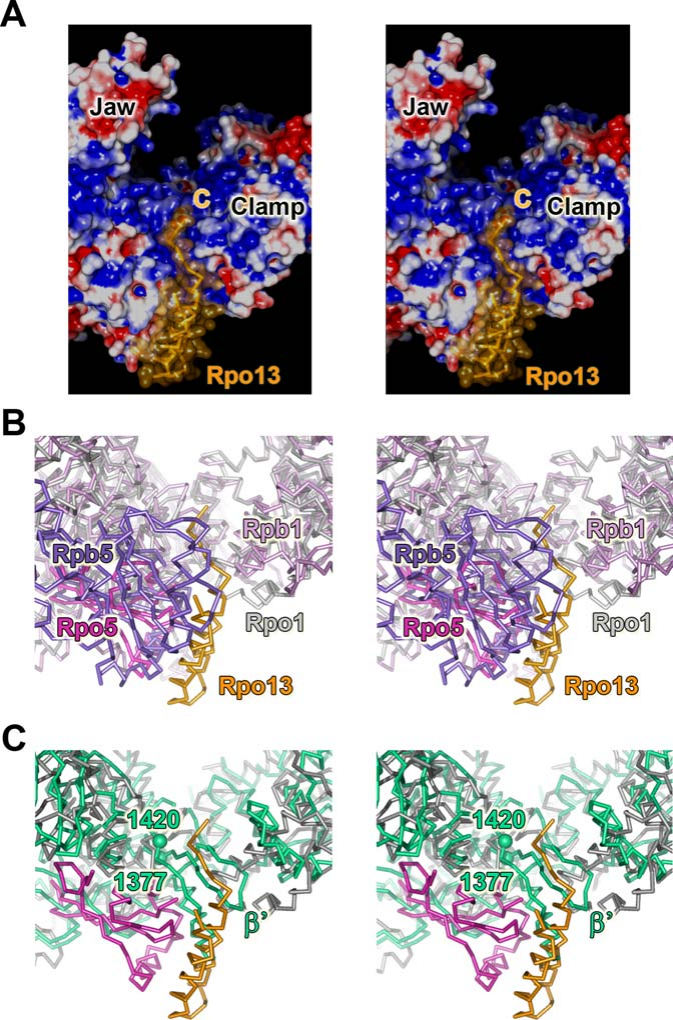
Binding *locus* of Subunit Rpo13 (A) Electrostatic surface representation (blue positive, red negative, and white neutral) with Rpo13 as orange ribbon (C labels the C-terminus) and semitransparent surface. (B) Stereo view of the docking region of Rpo13 (orange), in light-magenta Rpo5 and in grey Rpo1 with the eukaryotic Rpb5 (violet) and Rpb1 (plum; PDB entry 1WCM) superimposed; rmsd 2.4 Å for 1,129 Cα equivalences. (C) Archaeal Rpo13, Rpo5, and Rpo1 as (B) with the bacterial β′ from the T. aquaticus RNAP core structure (turquoise; PDB entry 1I6V) superimposed; rmsd 3.4 Å for 748 Cα equivalences. The two spheres mark the insertion region 1,377–1,420 in the bacterial β′ that spatially correlates with the archaeal subunit Rpo13. Superimpositions were carried out using the Structure Homology Program [44].

The structure determination in two distinct crystal forms, to 3.35 Å resolution is described in Materials and Methods; 3,334 residues in 13 subunits are seen, but the resolution achieved limits the accuracy of the position of the side chains. For the most flexible subunits, Rpo4 and Rpo7, accounting for some 265 residues, the register of the sequence is, in places, uncertain (±1). The model quality is good, it lies in the top 15th percentile of structures solved at between 3.1–3.6 Å as judged by MolProbity [11], 78% of the residues are in the most favoured region of the Ramachandran plot (see Table 1). This compares favourably with the equivalent value of 61% for the structure of RNAP from S. solfataricus [9]. The overall architecture, subunit arrangement, composition, and topology closely follow those of the eukaryotic counterpart RNAP II [2]; for this reason, we propose a new subunit nomenclature applicable to all archaeal RNAPs and based on the eukaryotic terminology (Figures 1 and 2). The basic assembly resembles (root mean square deviation [rmsd] 1.1 Å, for 2,938 residues aligned, 97% of the residues in common between the two structures) the recently published archaeal RNAP structure [9], but our structure adds considerable new information. More specifically, we have located the clamp-head domain in Rpo1N (Figure 2A), the jaw domain in subunit Rpo1C (Figure 2B), and the entire Rpo8 subunit (Figure 2C). Furthermore, we observe density corresponding to a helix-turn-helix (HTH) motif in a groove created by Rpo5 and the clamp-head domain of Rpo1N (Figures 2D and 3). Mass spectrometry analysis (see Materials and Methods) confirmed the presence of a previously reported RNAP subunit, named “component F” [12], comprising 104 residues of which the 45-residue HTH motif constitutes an ordered fragment. Fitting of the electron density yielded to a satisfactory alignment (Figures 2D and S1). We rename this subunit Rpo13, as RpoF has been used to refer to the distinct archaeal Rpb4 homolog. Uniquely in the archaeal RNAP, the Rpo13 subunit does not have an ortholog in the eukaryotic RNAP II.

Of the approximately 370-kDa archaeal RNAP, subunits Rpo1 (split into two subunits Rpo1N and Rpo1C) and Rpo2 represent more than two-thirds of the mass and are equivalent to bacterial β′ and β and to the eukaryotic Rpb1 and Rpb2 [9,13]. These subunits are composed of different domains that perform specific roles during RNA polymerisation in the active site of the Rpo1N subunit (catalytic residues D456, D458, and D460 are conserved across cellular RNAPs [2,8]). The second largest subunit, Rpo2, contains three Zn^2+^ atoms (two coordinated with His570 and with His696/His997, respectively; the third located in the clamp). Its polypeptide chain is largely ordered, and it provides, with Rpo1, the catalytic activity of the RNAP. Both subunits in the cellular RNAPs contain a double-Ψ β-barrel domain involved in the polymerization process, whose heterodimeric structure has been suggested as the ancestral core enzyme [13].

Subunits Rpo3 (containing a 4Fe-FS cluster [9]), Rpo6 and Rpo11 constitute, along with Rpo1 and Rpo2, the core RNAP, conserved across the three domains of life [8,9,13]. Structure-based phylogenetic analysis between the homologous components illustrates this evolutionary relationship (Figure S2) and strengthens the idea of a transcription apparatus that has increased cellular specificity associated with the addition of new functional modules. The remaining known archaeal subunits Rpo4/7, Rpo5, Rpo10, and Rpo12, with homologs only in Eukarya (class II subunits), decorate the core enzyme as shown in Figure 1 (Rpo10 and Rpo12 each bind a Zn^2+^). Of all the subunits common to Archaea/Eukarya, Rpo5/Rpb5 differs the most in size. Rpb5 is composed of a jaw and assembly domain [2], of which only the latter is present in the archaeal Rpo5 (which lacks the first ∼130 residues of Rpb5). The absence of the jaw domain allows access to a positively charged groove between the assembly domain and helices 3 and 4 of the clamp-head domain (see below and Figure 3A and 3B).

**Figure 4:**
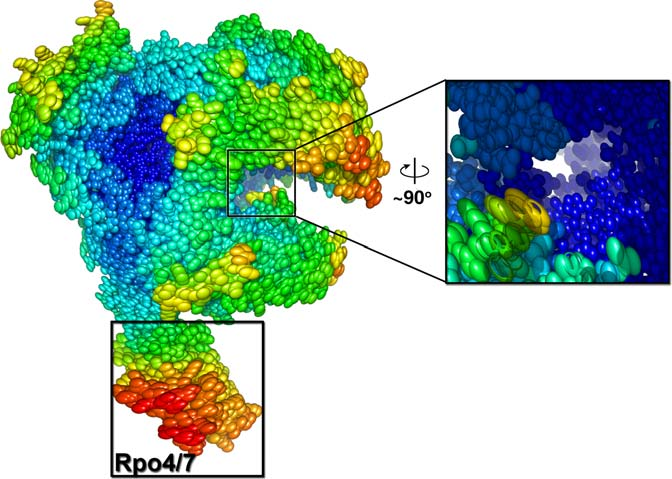
Ellipsoid Representation of the Thermal Motions of the Entire RNAP B ranges from Bmin = 40 Å^2^ (dark-blue) to Bmax = 233 Å^2^ (red) values obtained by normal mode analysis [27]. Subunits Rpo4/7 are highly mobile, whereas the inset highlights the catalytic site as a rigid frame. All figures in this article were generated in Pymol [45].

The Rpo4/7 heterodimer, which is conditionally required for initiation [14], protrudes from the main structure interacting mostly through Rpo6 and the C-terminus of Rpo1C (Figure 1). The Rpo4/7 stalk is highly mobile as judged by the electron density and normal mode analysis (Figure 4). Some weak density attributable to the C-terminus of subunit Rpo4 is visible and reminiscent of the position of the C-terminal helix 6 (H6) observed in the isolated Rpo4/7 crystal structure from Methanococcus jannaschii [15]. This suggests that H6 can move from its location, possibly contributing to the interaction with accessory cofactors such as transcription factor E (TFE) [7,8].

### Structural Conservation of the Flexible Clamp-Head and Jaw Domains of Rpo1 Subunit

The electron density maps reveal the clamp head domain (residues 97–173) in the Rpo1N subunit to be mostly ordered (residues 97–156). This domain, absent from the recent archaeal X-ray structure [9] is located at the DNA-binding cleft (Figure 2A), as is its eukaryotic counterpart. It adopts a fold similar to its eukaryotic ortholog and contains a Zn^2+^ ion chelated by C98, C101, and C146 (Figure 2A). It also shows some differences including a longer HTH motif (residues 107–140) that flexes by approximately 34°. This motif, together with the Rpo5 subunit, forms a cavity into which Rpo13 docks (Figure 3B and 3C). The remaining portion of the Rpo1N structure is well ordered, showing a Zn^2+^ ion in the clamp core domain and a single Mg^2+^ ion in the active site [2]. Electron density corresponding to the jaw domain of the Rpo1C subunit is also visible. This domain is implicated in binding DNA downstream of the transcription start site [4], and residues 147–236 have been modelled initially by reference to the eukaryotic polymerase (see Materials and Methods and Figure 2B). The bridge helix in Rpo1N and the trigger loop in Rpo1C, which have been proposed to play critical roles in DNA translocation [3], are respectively in a straight conformation (albeit with some differences from that observed by Hirata and co-workers [9] [rmsd 0.8 Å, for 40 aligned Cα]) and disordered. The location and the topology of the clamp-head and jaw domain in the Rpo1 subunit reinforce the structural similarity with Rpb1 of naked RNAP II.

### Structure of the Rpo8 Subunit

Until recently, it was believed that archaeal RNAPs from both the Crenarchaea and Euryarchaea kingdoms, the two main groups of Archaea, did not possess orthologs of Rpb8 [8,16], but recent work has identified divergent homologs of Rpb8 in the Crenarchaea [1,17]. This subunit, Rpo8 (15.1 kDa; 132 residues), is seen for the first time in our current study and is a constitutive structural element of the RNAP complex (Figures 1 and 2C). Rpo8 adopts an eight-stranded antiparallel β oligo-binding (OB) fold (Figure 2C) with the first three and last 15 residues disordered. It is located at a peripheral position (Figure 1), similar to eukaryotic Rpb8, with which it shares 112 Cα equivalences (out of 114) with 2.9 Å rmsd (Figure S3) and 14% sequence identity (the lowest sequence identity of all archaeal and eukaryotic subunits). Rpo8 interacts with subunit Rpo1N, sitting in the external crevice formed by residues 507–596 and burying a surface area of 1,470 Å^2^, equivalent to the interaction of Rpb8 with Rpb1 (Figure 2). The shorter Ω-loop motif (residues 63–69) is ordered, and the isosurface of electrostatic potential shows marked segregation of charges with a line of basic residues R20, K24, K52, K54, and K56 decorating the top of the molecule (Figure S4).

### Rpo13, a Novel Constituent RNAP Subunit

**Figure 5:**
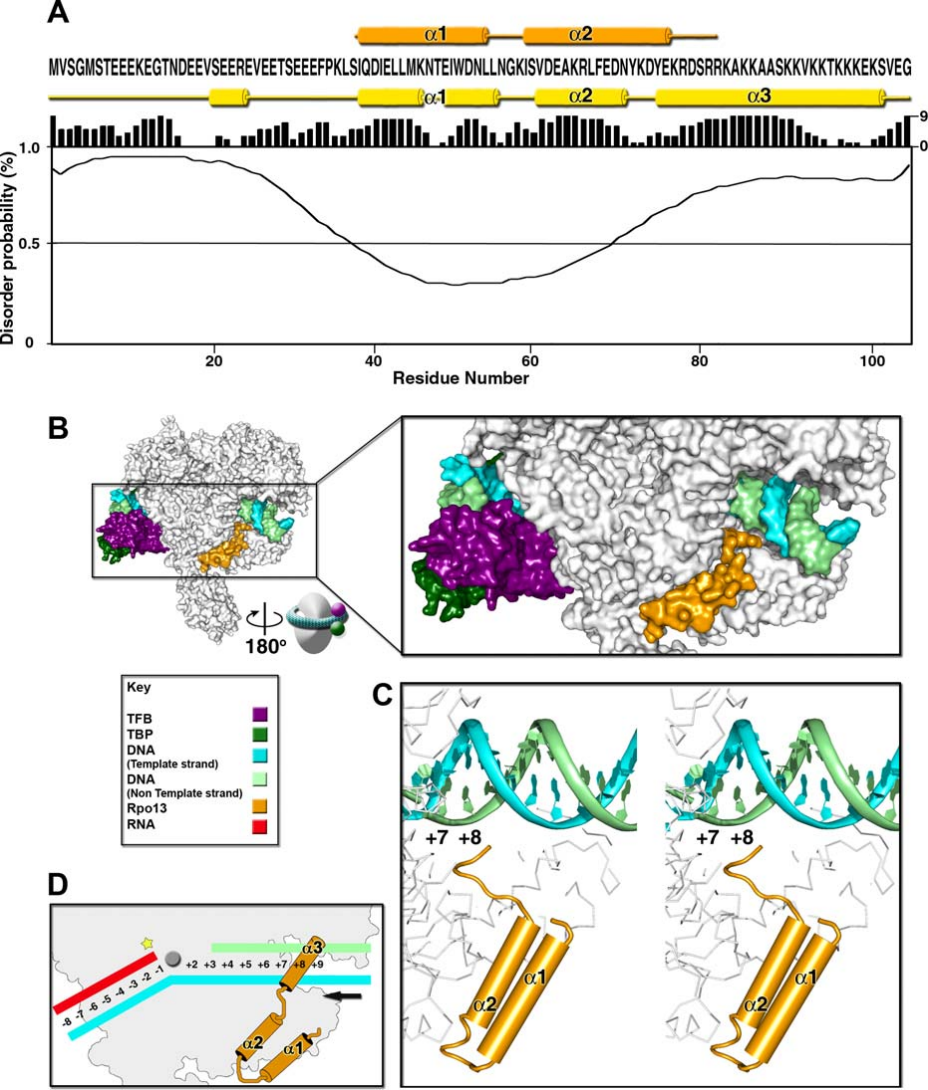
Archaeal Preinitiation Complex Model (A) Secondary structure elements of Rpo13 model (orange) aligned with the amino acid sequence (below), yellow secondary structure prediction (Protein Prediction [19]) with reliability histogram (0 = low to 9 = high), and graph of disorder prediction (RONN [18]). (B) Left, overall architecture with RNAP, TBP-TFB-DNA modelled as described in the main text, represented as a surface and coloured as in the key. Right, enlarged view of the TBP-TFB-DNA complex with the DNA wrapping the RNAP from the back (as viewed from left to right) with the downstream DNA in the entry channel close to Rpo13. (C) Stereo view of the two ordered helices (α1 and α2) of Rpo13 and DNA with the C-terminal end proximal to the DNA major groove at nucleotide +8. (D) Cartoon representation of the model of action of Rpo13 during polymerase activity (elements described in the key); star (yellow) marks the catalytic site, circle (grey) the bridge helix, and arrow (black) the direction of DNA translocation. The nucleic acid arrangement at the fork reflects the RNAP II elongation complex structure [46]. The location and orientation of Rpo13 α3 onto the DNA are speculative.

Predictions from the amino acid sequence [18,19] indicate an ordered HTH core motif (α1 and α2) with flexible N- and C-termini, respectively, predicted as mainly coil and α-helix (α3), suggesting a simple three-helix bundle protein prototypical of DNA binding proteins [20] with an N-terminal extension (Figure 5A).

The HTH motif of Rpo13 (absent from the recent model of the RNAP from S. solfataricus [9]), fits between Rpo5 and the clamp-head domain of Rpo1N (Figures 1 and 3) in the position equivalent to that occupied by residues 1377–1420, in the β′ large subunit of the crystal structures of the RNAP from the bacteria Thermus aquaticus and T. thermophilus RNAP [21,22] (Figure 3C). The corresponding sequence of this bacterial insertion is highly conserved within the Thermus-Deinococcus phylum, but there is also some detectable sequence similarity in other bacterial β′ sequences mainly of the Proteobacteria phylum. In T. aquaticus and *T. thermophilus*, this structure (whose function has not yet been characterized) folds into two antiparallel β strands followed by an α-helix; an organisation quite different from our Rpo13 (Figure 3C). In eukaryotes, the equivalent locus is partially occupied by the eukaryote-specific Rpb5 jaw domain (Figure 3B). Significantly, this domain is involved in downstream DNA binding [23], and the entire subunit has also been implicated in contacting transcription factor II B [24].

### RNAP Plasticity

We have used a normal-mode–based protocol [25] for structure refinement (see Material and Methods).

Although it is difficult to recognize biologically relevant modes per se, the principal modes present a simple dynamic picture of the RNAP, which might have relevance in vivo. The low-order modes include a pincer movement of the jaw-lobe module and clamp, in agreement with the structural variability found in static eukaryotic RNAP structures [2,26,27], and a contraction of the same structural elements generating a “ratchet” of the HTH motif and rudder (residues 278–297) in the clamp-core of Rpo1N; movements that can be detected by observing, for example, modes 2, 28, and 29 given in Protocols S1 and S2. This conformational plasticity would facilitate the transition of the RNAP from the apo- to the DNA-bound form for transcription initiation [2]. The Rpo4/7 subunits also swing along the side of the polymerase, a flexibility underlining the multiple roles played by these subunits [8]. This analysis defines the catalytic site as a rigid ensemble relative to the rest of the structure (Figure 4), providing enzymatic precision at the heart of a flexible machine [28].

### A Model for the Archaeal Preinitiation Complex

To gain insights into the assembly of the archaeal preinitiation complex, we have docked our intact RNAP onto the DNA–RNA hybrid visualized in the RNAP II transcribing complex [29], the eukaryotic RNAP-TFIIB complex [5], and the TFBc-TBP-DNA complex from Pyrococcus woesei [30] (TFB and TBP, respectively, share 51% and 46% sequence identity with their S. shibatae orthologs) (see Figures 5B and 5C and Text S2). The overall architecture of the archaeal preinitiation complex resembles the minimal initiating eukaryotic complex RNAP-TFIIB-TBP-DNA [5,31], but differs by not requiring homolog basal factors TFIIH and TFIIF and by the influence of TFE [6,8]. Our structure and the preinitiation complex model provide a rationale for this minimal set of cofactors (see Discussion below).

## Discussion

### Evolutionary Implications of the Newly Located RNAP Subunits

Our results clarify the evolutionary relationships of Archaea with Eukarya and Bacteria. The finding that Rpo8 is an integral component of the *Sulfolobus* enzyme, together with the ordered clamp-head and jaw domains in Rpo1, underscores the fact that the crenarchaeal and eukaryotic RNAPs have conserved the same basic enzymatic platform even when the sequence identity is lower than 15%. This implies a closer structural ancestry between RNAPs from Crenarchaea and Eukaryotes. A major structural difference however is the presence of the carboxyl-terminal-domain (CTD) in eukaryotic Rpb1. This feature presumably represents a later evolutionary acquisition, acting as a bolt-on module that facilitates coordination of eukaryote-specific cotranscriptional processing events such as capping, splicing, and polyadenylation.

On the other hand, whereas archaeal RNAP structurally anticipates the enzymatic machinery of the eukaryotic systems, RNAPs from the order Sulfolobales (and others from the Crenarchaea kingdom) have acquired Rpo13, a subunit that is not present in Eukarya and that corresponds architecturally to an insertion into the bacterial β′ subunit of T. aquaticus and T. thermophilus. Sequence analysis [32] shows that the *Rpo13* gene has orthologs in the orders Sulfolobales and Desulfurococcales of the Crenarchaeota phylum, but not in Euryarchaea (Figure S5). We have detected Rpo13 as part of the RNAP in three Sulfolobus species: first in our structure from S. shibatae (in both crystal forms, see Materials and Methods and Figure S6), then in S. acidocaldarius [12] and finally in S. solfataricus where we have reanalyzed the electron density (Figure S7). These results indicate that Rpo13 constitutes a stable structural component of the enzyme. Furthermore, the topology and location of the ordered fragment of Rpo13 suggests a mechanism of action in the context of the preinitiation complex assembly model (see below).

### Putative Role of Rpo13 at Initiation and Elongation

Although the function of Rpo13 is still unknown, its location leads us to hypothesize roles at initiation and elongation. Rpo13 could facilitate transcription bubble formation once the archaeal preinitiation complex is formed (modelled as in Figure 5B and 5C) and the N-terminal domain of TFB has driven the DNA towards the RNAP active centre [5,33]. The visible C-terminus of Rpo13 is at about 7 Å distance from the phosphate group of the nucleotide at position +8 from the start site of the modelled non–template DNA strand (Figure 5C). This close juxtaposition combined with the prediction of Rpo13 being a basic trihelical bundle protein, reminiscent of DNA recognition proteins, suggests that the third predicted helix of Rpo13 may interact with DNA. Additionally, the fact that Rpo13 has ten lysines in the last 20 residues supports an interaction of the predicted C-terminal α3 helix with negatively charged DNA rather than with the positively charged DNA binding cleft of Rpo1 (Figures 3A, 5A, 5C, and 5D). At initiation, the α3 helix of Rpo13 could provide a lock point, against which the main body of the polymerase cleft can push or twist the DNA; during elongation these locking interactions would be overcome by the translocation forces but may still interfere with DNA duplex stability. In this manner, Rpo13 may perform some of the roles attributed to eukaryote-specific general transcription factors. In this view, the α1 and α2 helices of Rpo13 act as constitutive anchors onto the RNAP, whereas α3 confers additional functionality, prefiguring some of the capabilities of removable cofactors needed for eukaryotic initiation.

Moreover, the presence of Rpo13 illustrates how the ancestral core enzyme was modulated by incorporation of novel subunits, a process that in eukaryotes has led to the emergence of three distinct classes of nuclear RNAPs.

## Materials and Methods

### Production and purification of RNA polymerase from *S. shibatae.*

Production and purification of archaeal RNAP are described elsewhere [34]. Briefly, S. shibatae cells were grown in three steps of 4 d each to a final optical density at 600 nm (OD_600_) ≈ 3.0. This growth served as inoculum for the final large-scale cell growth, carried on for additional 4 d and to an OD_600_ ≈ 4.0. The RNA polymerase was purified from the cell pellet by dialysis, Q Sepharose chromatography, and Hi-Trap heparin column.

### Crystallization and data collection.

Archaeal RNAP crystals were initially obtained with a microbatch under oil technique (Hampton) using purified RNAP at approximately 7.0 mg/ml in 150 mM KCl, 100 mM SrCl_2_, 100 mM Na-Cacodylate (pH 6.5), and 12% PEG MME 5K. This initial condition was expanded using a hanging-drop vapour diffusion technique and several attempts were made to optimize the original fragile crystals by adding 1 mM Zn^2+^ to exploring different gradient concentration of PEG 20K. Different datasets were collected either from native or heavy-atom–soaked crystals. Useful data that contributed to the initial phasing came from two crystals, a native close to 3.5 Å and a W11 tungsten cluster soak close to 4.0 Å (unpublished data), belonging to *P*2_1_2_1_2_1_ space group (Crystal_1) with two RNAP complexes per asymmetric unit (AU). A second native dataset was later collected from a crystal in *P*2_1_2_1_2 space group (Crystal_2) with one RNAP molecule in the AU. This was obtained by adding 5% glycerol to the above crystallization conditions and diffracted to a resolution of about 3.35 Å. All crystals were flash-frozen and the collected datasets indexed, integrated, and scaled using HKL [35]. A summary of the native data collection statistics is shown in Table 1.

### Structure determination and refinement.

Initially, the Crystal_1 structure was solved to a resolution of 6 Å by first finding a molecular replacement locked-rotation function solutions for two RNAP molecules in the asymmetric unit (GLRF program [36]), and subsequently by finding a phase translation function solution (MOLREP [37]) utilizing low-resolution phases obtained experimentally from crystals soaked in W11 tungsten clusters (SIRAS technique, SOLVE program [38]). Yeast RNAP polyalanine coordinates (Protein Data Bank [PDB] entry 1EN0) were used as a search model. The phases to approximately 4 Å were then 2-fold averaged and solvent flattened using DM [37] allowing the initial manual rebuilding of the different subunits as alanine models. When the data from the crystal in *P*2_1_2_1_2 space group (Crystal_2) were obtained, cross-crystal averaging between the two crystal forms was performed in GAP (unpublished program, D. I. Stuart and J. Grimes). Prior to this, rigid-body refinement of the initial polyalanine model was carried out in both crystal forms using REFMAC [37] and the correspondent 2Fo-Fc and Fo-Fc maps inspected for integrity of RNAP. The cross-averaged electron density map at 3.52 Å confirmed the presence of all subunits, including the Rpo8 and the additional density resembling a HTH motif close to Rpo1N (residues 97–151).

The sequence for each archaeal subunit was determined by PCR-mediated cloning and sequencing (see below) and docked onto the RNAP polyalanine model. The model was then improved by iterative manual building and refinement as described in detail in Text S1.

When the coordinates of the RNAP structure from S. solfataricus (PDB entry 2PMZ) [9] became available, the refinement process accelerated, and we turned our attention to the higher resolution data (3.35 Å; Crystal_2) encouraged by its higher signal-to-noise ratio at 3.52 Å (2.5 vs. 2.0 of Crystal_1) and confident of the correct but incomplete new starting model (PDB entry 2PMZ).

We finalized the refinement of our RNAP structure against the data collected from the *P*2_1_2_1_2 crystal by iterative manual adjustment of the model, normal-mode [25] and positional with B overall refinement in REFMAC [37]. A summary of the final statistics for Crystal_2 is shown in Table 1. As a validation tool, we extended the refinement of the Crystal_1 RNAP model whose current statistics are also included in Table 1.

All major findings for the RNAP structure in the *P*2_1_2_1_2 space group are observed in the *P*2_1_2_1_2_1_ structure including the presence of the Rpo13 subunit bound to the two RNAP molecules in the asymmetric unit (Figure S6). Coordinates and structure factors for both crystal forms have been deposited in the Protein Data Bank under ID codes 2WB1 (Crystal_1) and 2WAQ (Crystal_2).

### Sequencing of RNA polymerase subunits.

Open reading frames for the S. shibatae RNAP subunits were amplified by PCR from genomic DNA using Pfu DNA polymerase prior to cloning in PCR script (Stratagene) and sequencing (Geneservice). Primers were designed based on the conservation of flanking genes in S. solfataricus and S. tokodaii and are listed in Table S1. Table S2 shows the sequence identity with *S. solfataricus.*

### Identification of subunit Rpo13.

Inspection of the electron density of neighbouring subunits and superimposition with known RNAP components excluded the 2Fo-Fc electron density map correspondent to the unknown HTH motif of being an ordered or cleaved fragment of an already identified RNAP subunit. This structural element is reminiscent of the HTH domain of basal and specific transcription factors including the archaeal TFE [39] and DNA-binding proteins [20,40]. However, biochemical data rule out the possibility that this represents a fragment of TFE (unpublished data) and its binding *locus* is inconsistent with it belonging to transcription factor S (TFS) [4] or TFB [5]. To identify the novel archaeal subunit, we carried out liquid chromatography-mass spectroscopy with peptide fingerprinting [LC MS/MS; (HCTplus; Bruker Daltonics) coupled to a HPLC system (Ultimate; Dionex/LC-Packings)] of the sample used for crystallization at the Central Proteomics Facility Headington (CPF, University of Oxford). Using the sequenced genome of S. solfataricus [41] through an in-house Mascot server [42] (Matrixscience), we were able to fingerprint a protein component rich in lysine residues (Uniprot: SSO0396) corresponding to a previously detected RNAP component of the 13-subunit RNAP from S. acidocaldarius [12]. This gene is cotranscribed with *pcna3* (a DNA replication accessory factor), a situation reminiscent of the clustering of genes of other Rpos and information processing components [43].

The peptide mapping was carried out on the trypsin-digested fractions corresponding to molecular weight bands between 5 and 25 kDa extracted from a SDS-polyacrylamide gradient gel electrophoresis of the RNAP used for crystallization. The 2Fo-Fc electron density supported a protein binding to the RNAP in a 1:1 ratio and the packing restraints suggested a molecule no larger than approximately 250 residues. The gene for the homologous protein in S. shibatae was then sequenced and used for secondary and disorder structure predictions and model building (Figure 5A). The data search was rerun against our S. shibatae sequence database, and Figure S8 shows the Rpo13 peptides identified and the sequence coverage.

## Supporting Information

Figure S1 Stereo View of 2Fo-Fc and Fo-Fc Electron Density Maps of Rpo13 Stereo view of 2Fo-Fc (blue, contoured at 0.9σ) and Fo-Fc (red, contoured at 2.5σ) sigmaA-weighted maps prior the assignment of the Rpo13 sequence onto the poly-alanine helix-turn-helix model (as stick orange) in the RNAP structure of the crystal in *P*2_1_2_1_2 space group. The black circle highlights the density corresponding to the residue used as a marker for sequence assignment**.**
(1.26 MB DOC)

Figure S2 Structure-Based Phylogenetic Trees of the Core Conserved Subunits in Archaea, Eukarya, and Bacteria Structures were generated using a modified version of the Structure Homology Program [47] and PHYLIP package [48]. At the centre the surface representation of the archaeal core enzyme. Nomenclature as Figure 1.
(1.33 MB DOC)

Figure S3 Stereo View of Superimposed Ca Traces of Archaeal Rpo8 and Eukaryotic Rpb8 Stereo view of superimposed Cα traces of archaeal Rpo8 (red ribbon) and eukaryotic Rpb8 (yellow ribbon; PDB entry 1I50); N and C label respectively the N terminus and C terminus of Rpo8.
(799 KB DOC)

Figure S4 Comparison of Surface Charge Distribution of Archaeal Rpo8 versus Eukaryotic Rpb8 Comparison of the surface charge distribution (blue positive, red negative and white neutral) of archaeal Rpo8 versus*.* the eukaryotic Rpb8 viewed accordingly to ssDNA binding surfaces of Rpb8 [49]. The left panel is related to the Rpo8 viewed in Figure 2A by 90° rotation, and the right panel by a further 90° as indicated by the central arrow. (A) Electrostatic potential isosurface of Rpo8 with the positions of basic residues lining on the crest of the molecule (K56 not visible). (B) Rpb8 potential surface.
The charges assignments were generated with PDB2PQR program using Amber charges [50].
(2.77 MB DOC)

Figure S5 Multiple Sequence Alignment of Rpo13 Orthologs in Sulfolobales and Desulfurococcales Multiple sequence alignment (ClustalW [51] and Espript [52]) between the protein sequences of Rpo13 in *Sulfoloboles* and *Desulfurococcales*. Sulfolobus solfataricus (gene: SSO0396), Sulfolobus todakaii (gene: ST0398), Sulfolobus acidolcadarius (gene: Saci_0816), Metallosphaera sedula (gene: Msed_0052), and *Staphylothermus marinus F1* (gene: Smar_1004).
(920 KB DOC)

Figure S6 Stereo Views of 2Fo-Fc Electron Density Maps of the Two Independent Rpo13 Subunits of the Crystal in *P*2_1_2_1_2_1_ Space Group Stereo views of the two independent Rpo13 subunits (as orange Cα trace) within the asymmetric unit of the crystal in *P*2_1_2_1_2_1_ space group with correspondent 2Fo-Fc maps (contoured at 0.9σ) calculated from the current model (see Crystal_1 refinement in Table 1). Both Rpo13 molecules slightly differ by having less ordered N- and C- terminal extensions than the Rpo13 modelled in the *P*2_1_2_1_2 crystal, a situation contributed by the different packing environment constraints that supports the prediction of a more flexible N- and C-terminal domains (Figure 5A).
(1.03 MB DOC)

Figure S7 Stereo View of 2Fo-Fc and Fo-Fc Electron Density Maps of Rpo13 Corresponding Regions in the RNAP from S. solfataricus
Stereo view of 2Fo-Fc and Fo-Fc electron density maps calculated via EDS in Coot [53] from the PDB entry 2PMZ corresponding to the structure of the RNAP from Sulfolobus solfataricus [9] and contoured respectively at 0.9σ (blue) and 2.3σ (positive green; negative red) of the corresponding region where we have found Rpo13 in our RNAP. Two side-by-side rod-like shaped densities are clearly visible packing against Rpo5 (as yellow ribbon). Density was also observed for subunit Rpo8 in Sulfolobus solfataricus RNAP structure [9] (unpublished data).
(1.50 MB DOC)

Figure S8 Mascot Data Results for Rpo13 Mascot data results for Rpo13 (S. shibatae) from LC MS/MS experiment. On top, the sequence coverage (63%; red) obtained by identification of the fingerprinted peptides generated by trypsinization of gel fractions.
(1.17 MB DOC)

Protocol S1 Modes from 1 to 15 Correspond to DCD Files Labelled ca#.dcd Where # Corresponds to the Mode Number To visualize the motions of the individual normal modes, the Cα pdb coordinate file (labelled ca.pdb) and the mode of interest can be uploaded in the VMD program (http://www.ks.uiuc.edu/Research/vmd/).
(5.79 MB ZIP)

Protocol S2 Modes from 16 to 30 Correspond to DCD Files Labelled ca#.dcd Where # Corresponds to the Mode Number To visualize the motions of the individual normal modes, the Cα pdb coordinate file (labelled ca.pdb) and the mode of interest can be uploaded in the VMD program (http://www.ks.uiuc.edu/Research/vmd/).
(5.76 MB ZIP)

Protocol S3 Modes from 31 to 50 Correspond to DCD Files Labelled ca#.dcd Where # Corresponds to the Mode Number To visualize the motions of the individual normal modes, the Cα pdb coordinate file (labelled ca.pdb) and the mode of interest can be uploaded in the VMD program (http://www.ks.uiuc.edu/Research/vmd/).
(7.68 MB ZIP)

Table S1 Primers for Sequencing of S. shibatae RNAP Subunits (39 KB DOC)

Table S2 Sequence Identity to S. solfataricus Ortholog (44 KB DOC)

Text S1 Supplementary Methods: Model Building and Refinement Protocol (41 KB DOC)

Text S2 Supplementary Methods: Modelling of the Preinitiation Complex (32 KB DOC)

## Abbreviations

HTH: helix-turn-helix
rmsd: root mean square deviation
RNAP: RNA polymerase
TBP: TATA-box binding protein
TFB: transcription factor B
TFE: transcription factor E
TFIIS: transcription factor II S
TFIIB: transcription factor II B
