# Correction: Evolution of Complex RNA Polymerases: The Complete Archaeal RNA Polymerase Structure

**DOI:** 10.1371/annotation/042f2803-a625-4b2a-bd08-ce50799e4cf6

**Published:** 2010-08-20

**Authors:** Yakov Korkhin, Ulug M Unligil, Otis Littlefield, Pamlea J Nelson, David I Stuart, Paul B Sigler, Stephen D Bell, Nicola G. A Abrescia

In Text S2, the references are missing. Please view the correct Text S2 here: 
