# Supplementary material for: Evolution of Complex RNA Polymerases: The Complete Archaeal RNA Polymerase Structure
Source: PLoS Biol. 2009 May 5;7(5):e1000102. doi: 10.1371/journal.pbio.1000102 (PMC2675907; doi:10.1371/journal.pbio.1000102)
Supplement: Figure S1 — Stereo view of 2Fo-Fc (blue, contoured at 0.9σ) and Fo-Fc (red, contoured at 2.5σ) sigmaA-weighted maps prior the assignment of the Rpo13 sequence onto the poly-alanine helix-turn-helix model (as stick orange) in the RNAP structure of the crystal in P21212 space group. The black circle highlights the density corresponding to the residue used as a marker for sequence assignment. (1.26 MB DOC) [file pbio.1000102.sg001.doc]

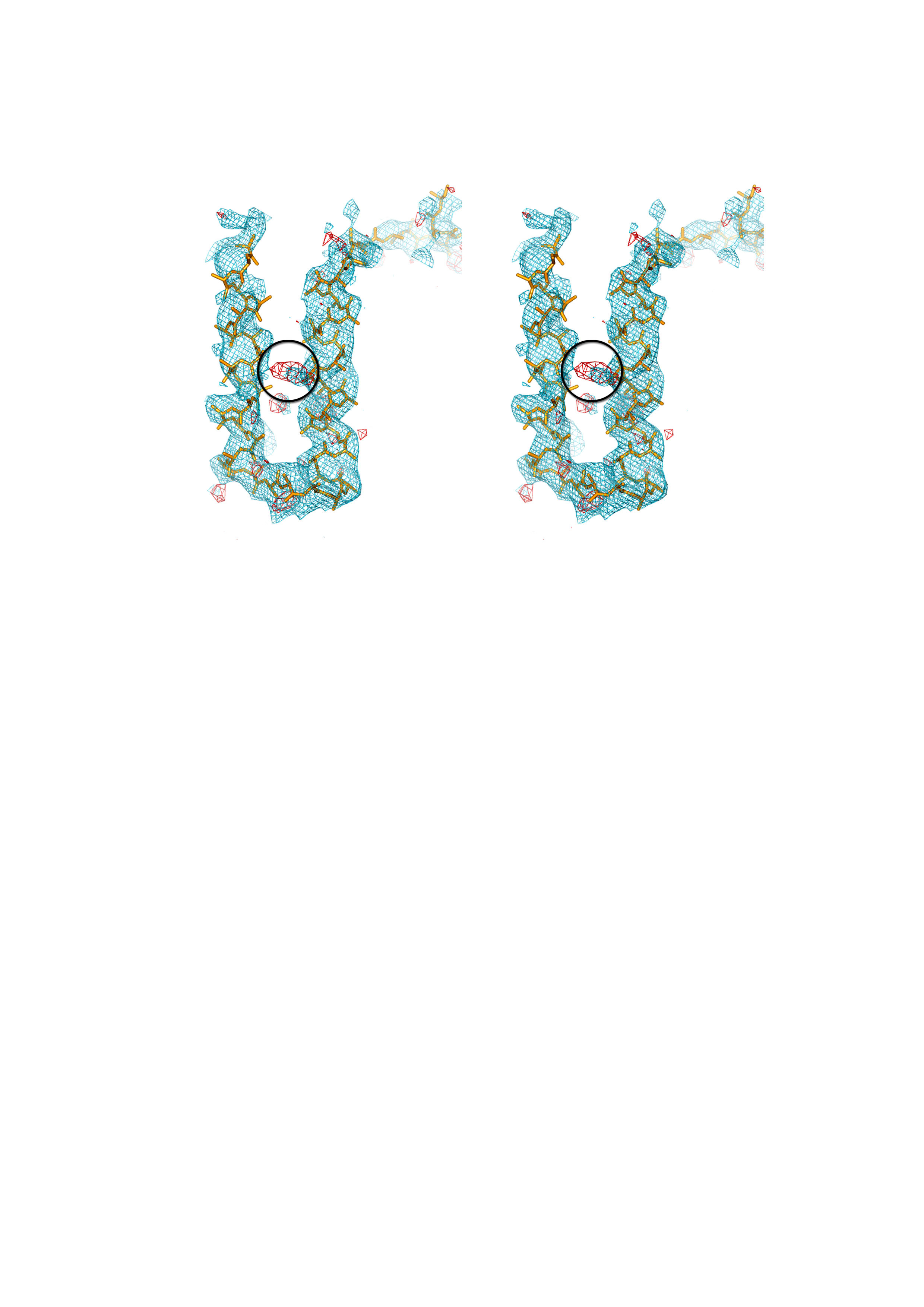


**Figure S1** Stereo view of 2Fo-Fc (blue, contoured at 0.9σ) and Fo-Fc (red, contoured at 2.5σ) sigmaA-weighted maps prior the assignment of the Rpo13 sequence onto the poly-alanine helix-turn-helix model (as stick orange) in the RNAP structure of the crystal in *P*2_1_2_1_2 space group. The black circle highlights the density corresponding to the residue used as a marker for sequence assignment**.**
