# Supplementary material for: Evolution of Complex RNA Polymerases: The Complete Archaeal RNA Polymerase Structure
Source: PLoS Biol. 2009 May 5;7(5):e1000102. doi: 10.1371/journal.pbio.1000102 (PMC2675907; doi:10.1371/journal.pbio.1000102)
Supplement: Figure S2 — Structures were generated using a modified version of the Structure Homology Program [47] and PHYLIP package [48]. At the centre the surface representation of the archaeal core enzyme. Nomenclature as Figure 1. (1.33 MB DOC) [file pbio.1000102.sg002.doc]

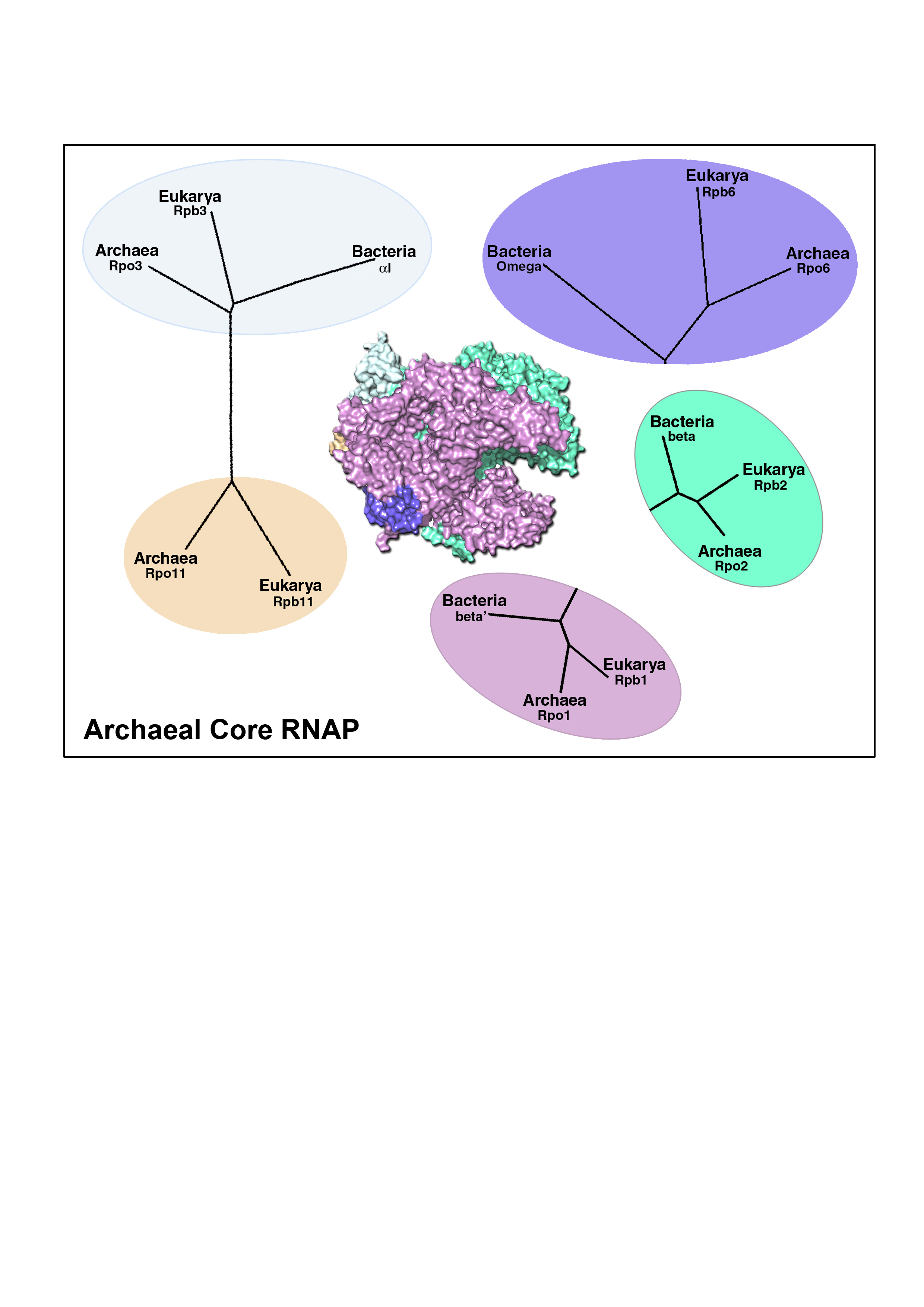
**Figure S2** Structure-based phylogenetic trees of the core conserved subunits in Archaea, Eukarya and Bacteria (nomenclature as Figure 1) generated using a modified version of the Structure Homology Program [47] and PHYLIP package [48]. At the centre the surface representation of the archaeal core enzyme.
