# Supplementary material for: Evolution of Complex RNA Polymerases: The Complete Archaeal RNA Polymerase Structure
Source: PLoS Biol. 2009 May 5;7(5):e1000102. doi: 10.1371/journal.pbio.1000102 (PMC2675907; doi:10.1371/journal.pbio.1000102)
Supplement: Figure S3 — Stereo view of superimposed Cα traces of archaeal Rpo8 (red ribbon) and eukaryotic Rpb8 (yellow ribbon; PDB entry 1I50); N and C label respectively the N terminus and C terminus of Rpo8. (799 KB DOC) [file pbio.1000102.sg003.doc]

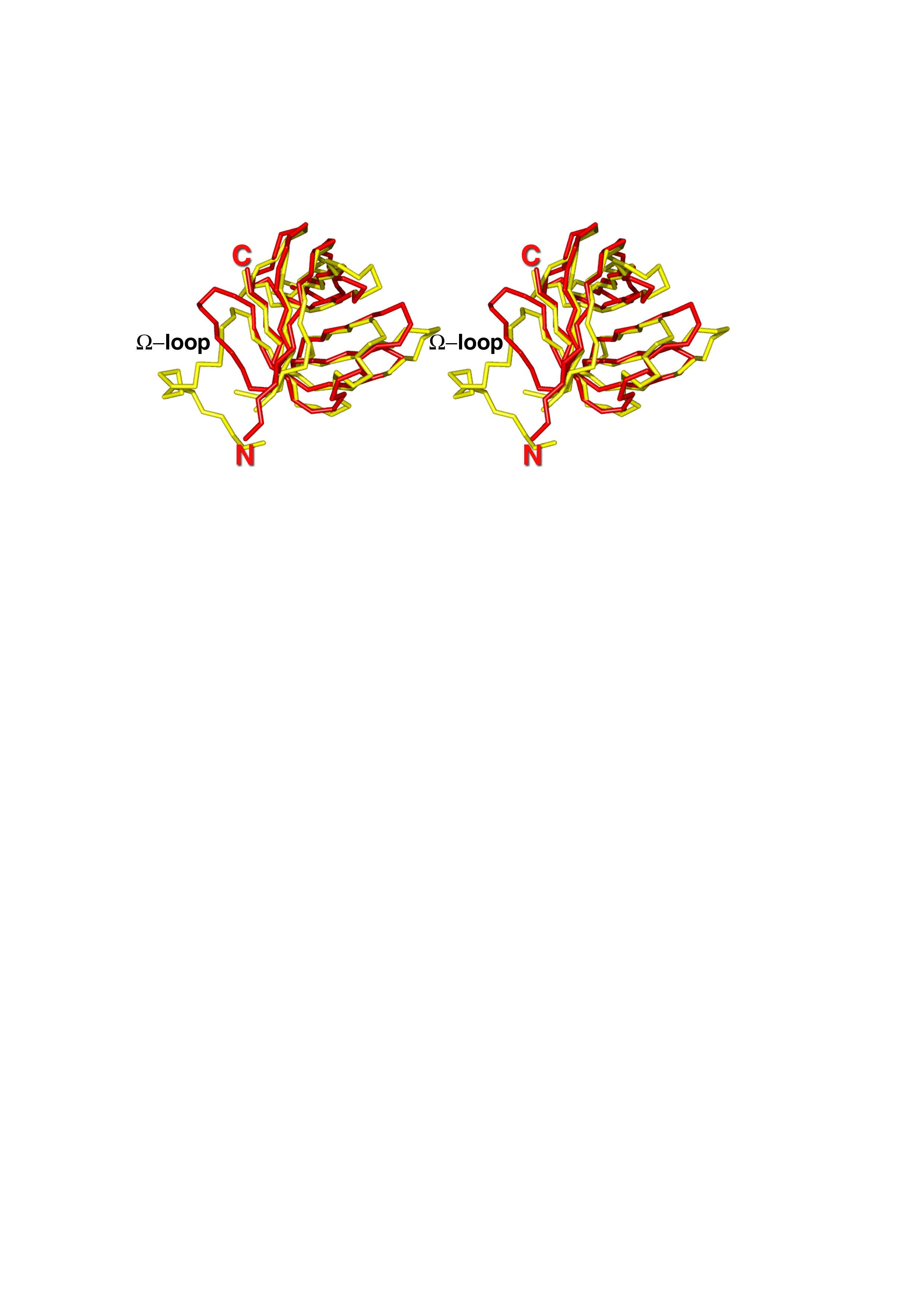


**Figure S3** Stereo-view of superimposed Cα traces of archaeal Rpo8 (red ribbon) and eukaryotic Rpb8 (yellow ribbon; PDB entry 1I50); N and C label respectively the N-terminus and C-terminus of Rpo8.
