# Supplementary material for: Evolution of Complex RNA Polymerases: The Complete Archaeal RNA Polymerase Structure
Source: PLoS Biol. 2009 May 5;7(5):e1000102. doi: 10.1371/journal.pbio.1000102 (PMC2675907; doi:10.1371/journal.pbio.1000102)
Supplement: Figure S4 — Comparison of the surface charge distribution (blue positive, red negative and white neutral) of archaeal Rpo8 versus. the eukaryotic Rpb8 viewed accordingly to ssDNA binding surfaces of Rpb8 [49]. The left panel is related to the Rpo8 viewed in Figure 2A by 90° rotation, and the right panel by a further 90° as indicated by the central arrow. (A) Electrostatic potential isosurface of Rpo8 with the positions of basic residues lining on the crest of the molecule (K56 not visible). (B) Rpb8 potential surface. The charges assignments were generated with PDB2PQR program using Amber charges [50]. (2.77 MB DOC) [file pbio.1000102.sg004.doc]

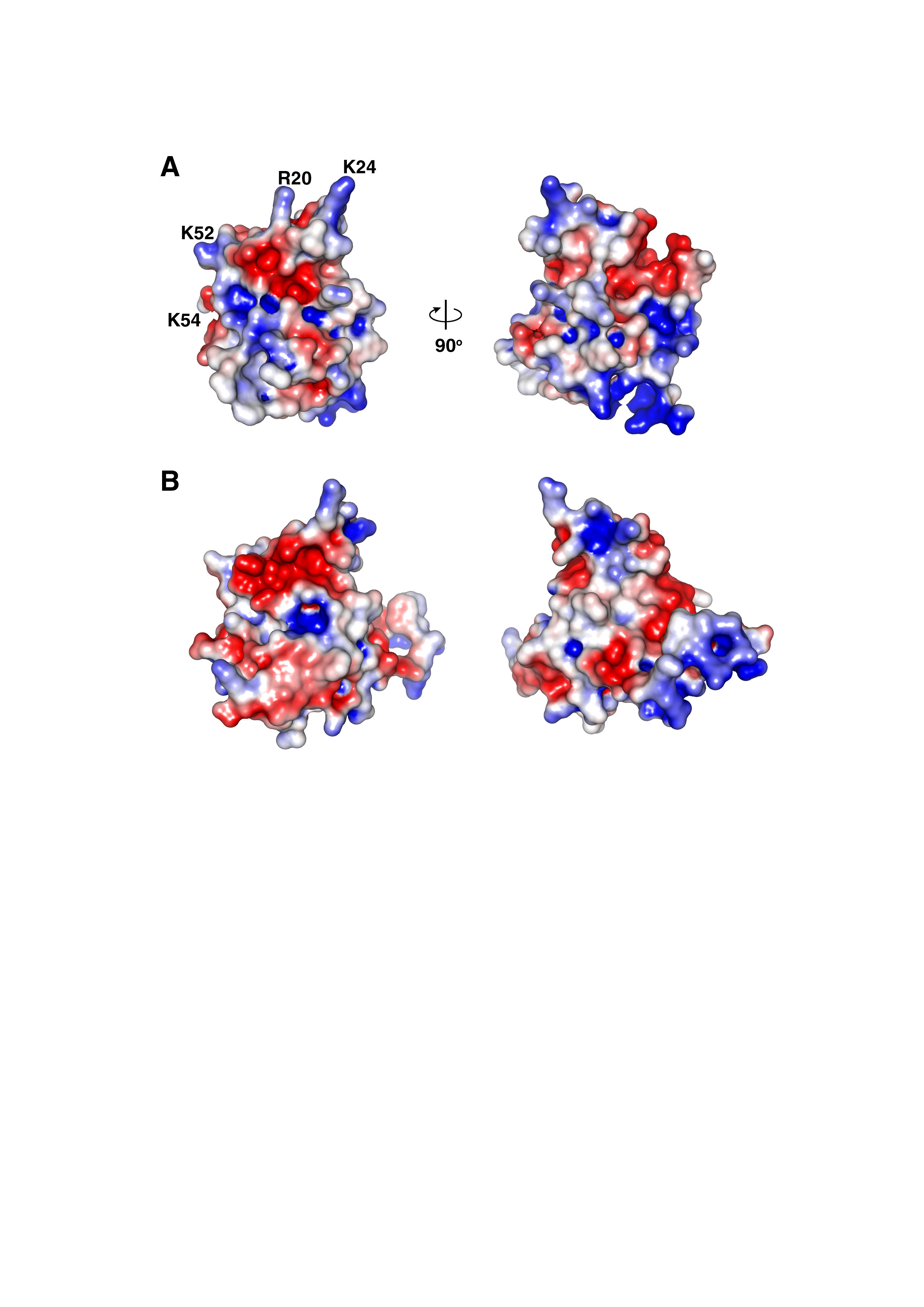


**Figure S4** Comparison of the surface charge distribution (blue positive, red negative and white neutral) of archaeal Rpo8 *vs.* the eukaryotic Rpb8 viewed accordingly to ssDNA binding surfaces of Rpb8 [49]. The left panel is related to the Rpo8 viewed in Fig. 2A by 90^o^ rotation and the right panel by a further 90 degrees as indicated by the central arrow. (**A**) Electrostatic potential isosurface of Rpo8 with the positions of basic residues lining on the crest of the molecule (K56 not visible). (**B**) Rpb8 potential surface.

The charges assignments were generated with PDB2PQR program using Amber charges [50].
