# Supplementary material for: Evolution of Complex RNA Polymerases: The Complete Archaeal RNA Polymerase Structure
Source: PLoS Biol. 2009 May 5;7(5):e1000102. doi: 10.1371/journal.pbio.1000102 (PMC2675907; doi:10.1371/journal.pbio.1000102)
Supplement: Figure S5 — Multiple sequence alignment (ClustalW [51] and Espript [52]) between the protein sequences of Rpo13 in Sulfoloboles and Desulfurococcales. Sulfolobus solfataricus (gene: SSO0396), Sulfolobus todakaii (gene: ST0398), Sulfolobus acidolcadarius (gene: Saci_0816), Metallosphaera sedula (gene: Msed_0052), and Staphylothermus marinus F1 (gene: Smar_1004). (920 KB DOC) [file pbio.1000102.sg005.doc]

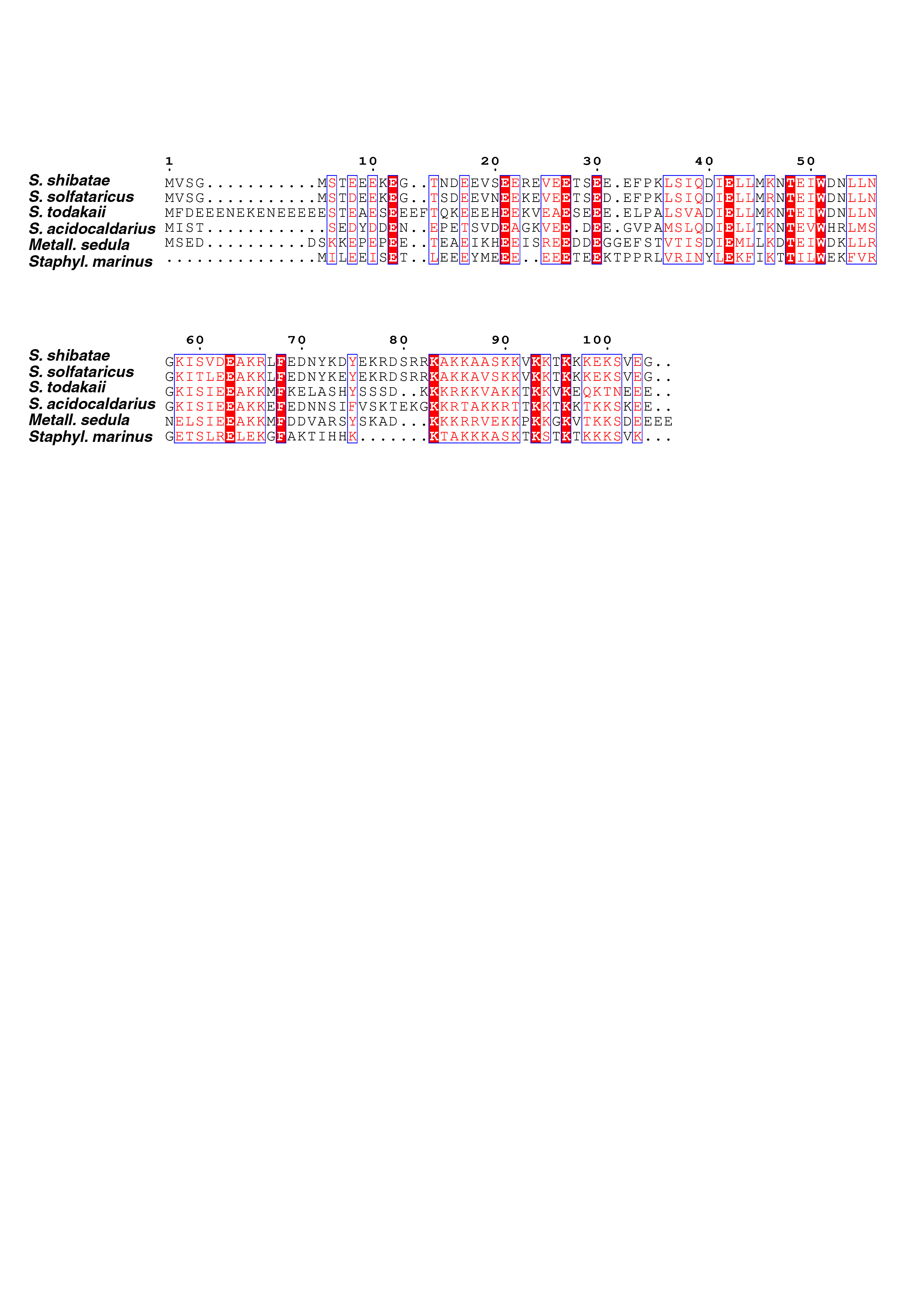
**Figure S5** Multiple sequence alignment (ClustalW [51] and Espript [52]) between the protein sequences of Rpo13 in *Sulfoloboles* and *Desulfurococcales*. *Sulfolobus solfataricus* (gene: SSO0396), *Sulfolobus todakaii* (gene: ST0398), *Sulfolobus acidolcadarius* (gene: Saci_0816), *Metallosphaera sedula* (gene: Msed_0052), *Staphylothermus marinus F1* (gene: Smar_1004).
