# Supplementary material for: Evolution of Complex RNA Polymerases: The Complete Archaeal RNA Polymerase Structure
Source: PLoS Biol. 2009 May 5;7(5):e1000102. doi: 10.1371/journal.pbio.1000102 (PMC2675907; doi:10.1371/journal.pbio.1000102)
Supplement: Figure S6 — Stereo views of the two independent Rpo13 subunits (as orange Cα trace) within the asymmetric unit of the crystal in P212121 space group with correspondent 2Fo-Fc maps (contoured at 0.9σ) calculated from the current model (see Crystal_1 refinement in Table 1). Both Rpo13 molecules slightly differ by having less ordered N- and C- terminal extensions than the Rpo13 modelled in the P21212 crystal, a situation contributed by the different packing environment constraints that supports the prediction of a more flexible N- and C-terminal domains (Figure 5A). (1.03 MB DOC) [file pbio.1000102.sg006.doc]

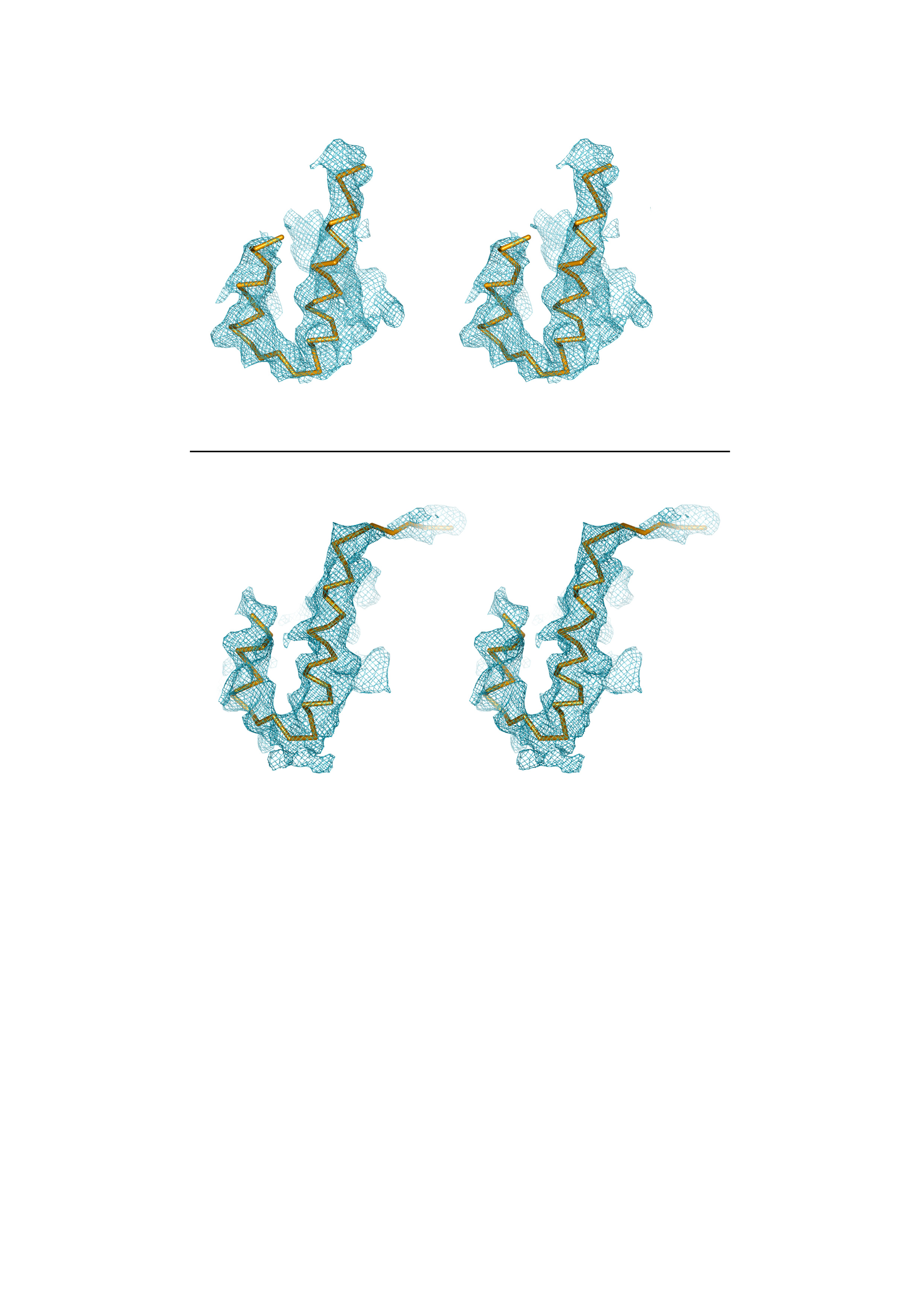


**Figure S6** Stereo views of the two independent Rpo13 subunits (as orange Cα trace) within the asymmetric unit of the crystal in *P*2_1_2_1_2_1_ space group with correspondent 2Fo-Fc maps (contoured at 0.9σ) calculated from the current model (see Crystal_1 refinement in Table 1). Both Rpo13 molecules slightly differ by having less ordered N and C- terminal extensions than the Rpo13 modelled in the *P*2_1_2_1_2 crystal, a situation contributed by the different packing environment constraints that supports the prediction of a more flexible N and C-terminal domains (Figure 4A).
