# Supplementary material for: Evolution of Complex RNA Polymerases: The Complete Archaeal RNA Polymerase Structure
Source: PLoS Biol. 2009 May 5;7(5):e1000102. doi: 10.1371/journal.pbio.1000102 (PMC2675907; doi:10.1371/journal.pbio.1000102)
Supplement: Figure S7 — Stereo view of 2Fo-Fc and Fo-Fc electron density maps calculated via EDS in Coot [53] from the PDB entry 2PMZ corresponding to the structure of the RNAP from Sulfolobus solfataricus [9] and contoured respectively at 0.9σ (blue) and 2.3σ (positive green; negative red) of the corresponding region where we have found Rpo13 in our RNAP. Two side-by-side rod-like shaped densities are clearly visible packing against Rpo5 (as yellow ribbon). Density was also observed for subunit Rpo8 in Sulfolobus solfataricus RNAP structure [9] (unpublished data). (1.50 MB DOC) [file pbio.1000102.sg007.doc]

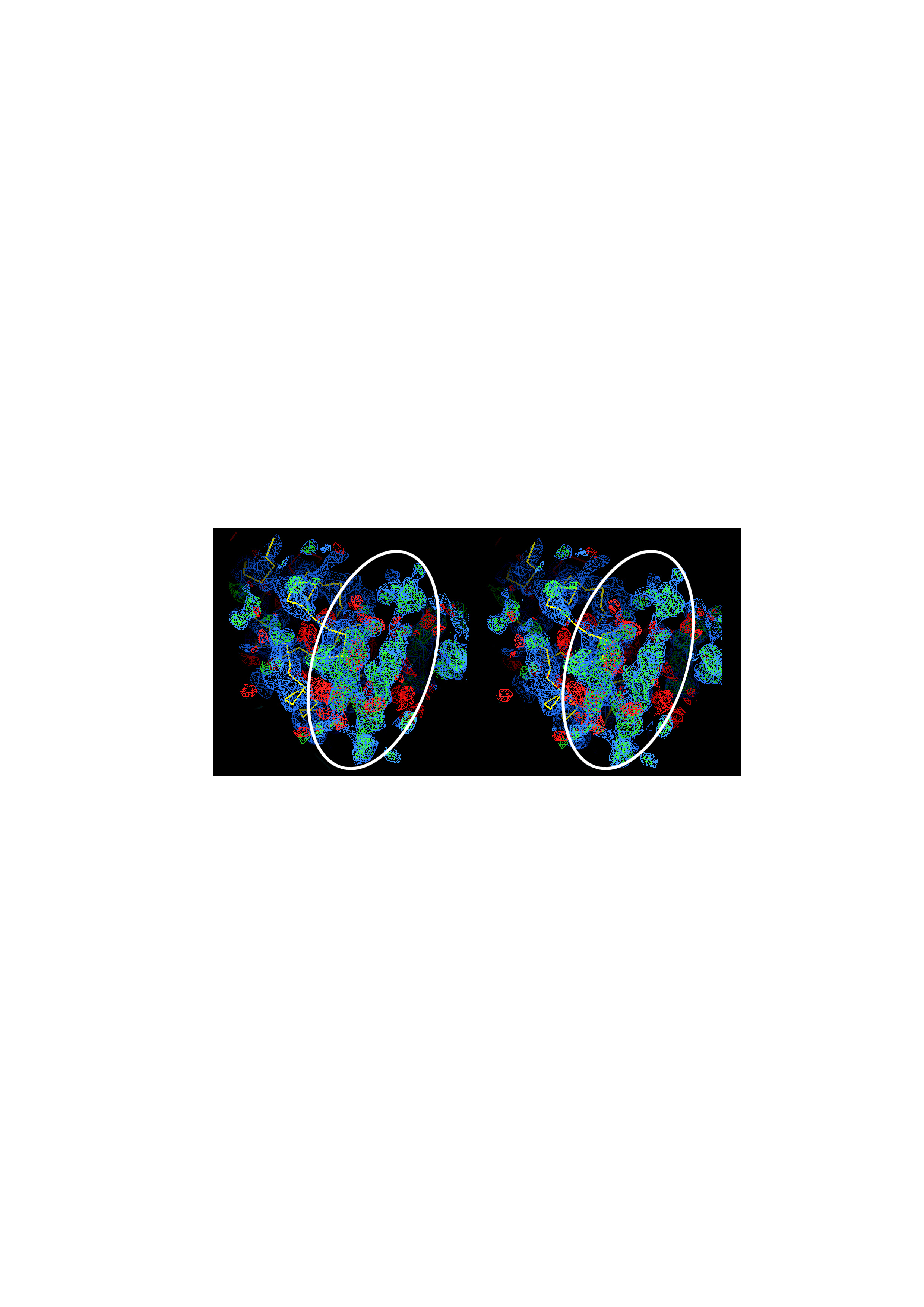


**Figure S7** Stereo view of 2Fo-Fc and Fo-Fc electron density maps calculated via EDS in Coot [53] from the PDB entry 2PMZ corresponding to the structure of the RNAP from *Sulfolobus solfataricus* [9] and contoured respectively at 0.9σ (blue) and 2.3σ (positive green; negative red) of the corresponding region where we have found Rpo13 in our RNAP. Two side-by-side rod-like shaped densities are clearly visible packing against Rpo5 (as yellow ribbon). Density was also observed for subunit Rpo8 in *Sulfolobus solfataricus* RNAP structure [9] (data not shown).
