# Supplementary material for: Evolution of Complex RNA Polymerases: The Complete Archaeal RNA Polymerase Structure
Source: PLoS Biol. 2009 May 5;7(5):e1000102. doi: 10.1371/journal.pbio.1000102 (PMC2675907; doi:10.1371/journal.pbio.1000102)
Supplement: Figure S8 — Mascot data results for Rpo13 (S. shibatae) from LC MS/MS experiment. On top, the sequence coverage (63%; red) obtained by identification of the fingerprinted peptides generated by trypsinization of gel fractions. (1.17 MB DOC) [file pbio.1000102.sg008.doc]

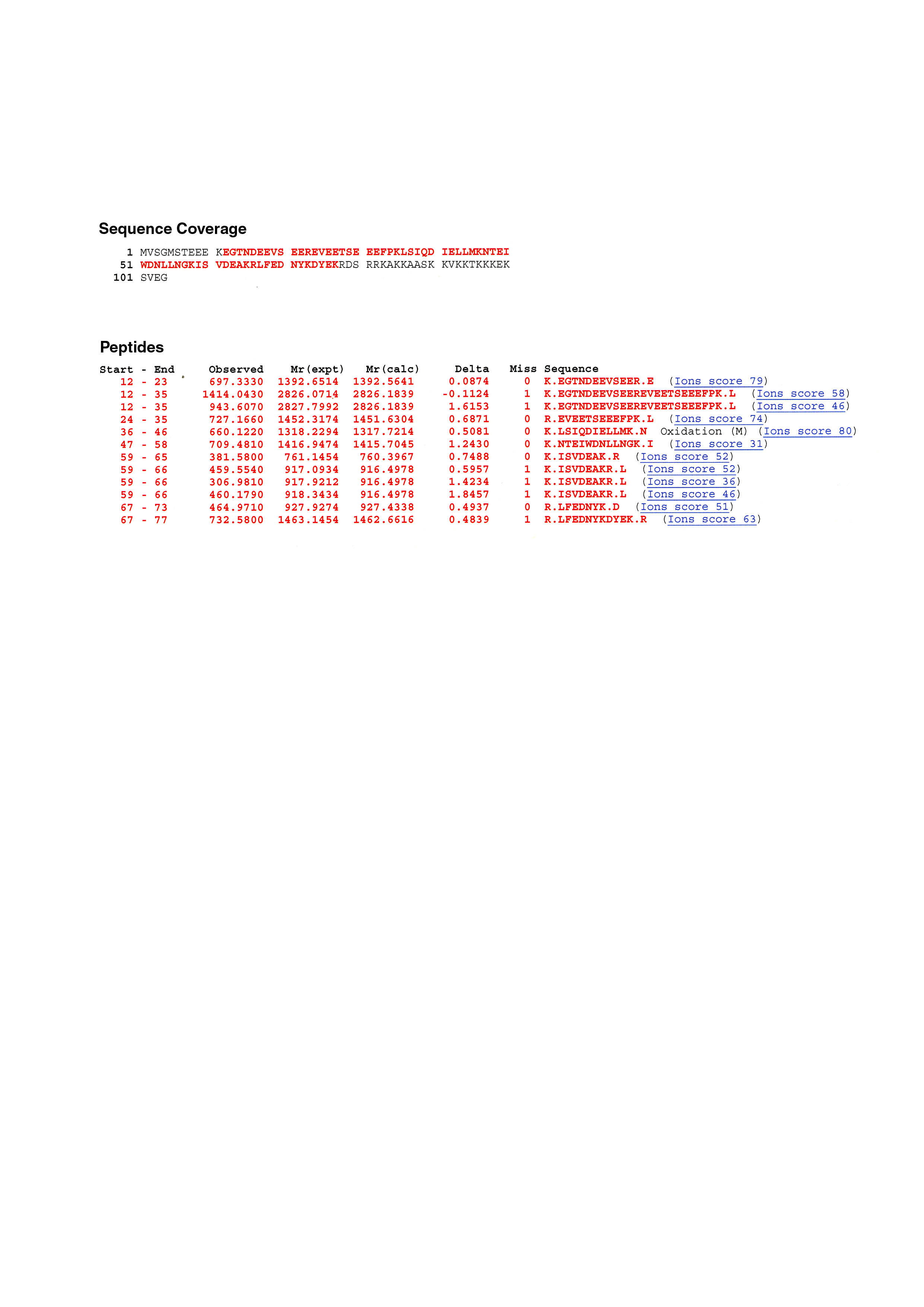
**Figure S8** Mascot data results for Rpo13 (*S. shibatae*) from LC MS/MS experiment. On top the sequence coverage (63%; red) obtained by identification of the fingerprinted peptides generated by trypsinization of gel fractions.
