# Supplementary material for: Evolution of Complex RNA Polymerases: The Complete Archaeal RNA Polymerase Structure
Source: PLoS Biol. 2009 May 5;7(5):e1000102. doi: 10.1371/journal.pbio.1000102 (PMC2675907; doi:10.1371/journal.pbio.1000102)
Supplement: Table S1 — (39 KB DOC) [file pbio.1000102.st001.doc]

**Table S1. Primers for sequencing of *S. shibatae* RNAP subunits**

| **Subunit** | **Primers**  **(Forward/Reverse)** |  |
| --- | --- | --- |
| Rpo1N, Rpo1C, Rpo2 and Rpo5 | CAAGTAATGTTGTTTTCCCATGATCTACATG /  AGAAATCATAAACGGTTTACCGCATA |  |
| Rpo3 and Rpo10 | TAAGGAGACCTGGCGGAAGAAGAGGAAGAAGAGT /  CCTACTTTAGCTGCTTCTTTTATWGCTTG |  |
| Rpo4 | TTRTCTAATGGATTACCCTSTCTCAWATAATCTAA /  ATAAATCCWTCWRTACATMAAGGTATGCCTCATAG |  |
| Rpo6 | CCTTCTATATCGTAATCNCANGCATTGATAAA /  TTAGGNAANGANANTAACTTTTCTAATAC |  |
| Rpo7 | GGAGANTNATAGGATGGGG /  TTNATAAAAAGAANNCCATATGG |  |
| Rpo8 | CAGCTAAAYTACTTTCRGTAATACTATACCA /  ATTTCCATTWATYATTAMCTTATAG |  |
| Rpo11 | ANGCAAGACGAANANNATTTAAAATG /  CCCGCCGGGATTTGAACCCGGGACC |  |
| Rpo12 | CTCAATAACTAGAATGTTAGATTCC /  TGATATGGATGGGATAGATCCAGC |  |
| Rpo13 | GGTAATTTACAAACTAT /  TCAATTTTCATAGGTATRTTWTYTCCAAA |  |
| (Y=T or C; M=A or C; R=A or G; W=A or T, S=G or C; N=any base) | | |
