# Supplementary material for: Evolution of Complex RNA Polymerases: The Complete Archaeal RNA Polymerase Structure
Source: PLoS Biol. 2009 May 5;7(5):e1000102. doi: 10.1371/journal.pbio.1000102 (PMC2675907; doi:10.1371/journal.pbio.1000102)
Supplement: Table S2 — (44 KB DOC) [file pbio.1000102.st002.doc]

**Table S2. Sequence identity (similarity) to *S. solfataricus* ortholog**

|  | **% identity (similarity)**  **to *S. solfataricus* ortholog** | **GenBank Accession Number** |
| --- | --- | --- |
| Rpo1N | 98 (99) | FJ515665 |
| Rpo1C | 94 (98) | FJ515666 |
| Rpo2 | 96 (99) | FJ515667 |
| Rpo3 | 91 (96) | FJ515668 |
| Rpo4 | 89 (97) | FJ515669 |
| Rpo5 | 97 (100) | FJ515672 |
| Rpo6 | 94 (98) | FJ515673 |
| Rpo7 | 98 (99) | FJ515670 |
| Rpo8 | 84 (93) | FJ515671 |
| Rpo10 | 93 (98) | FJ515675 |
| Rpo11 | 91 (95) | FJ515674 |
| Rpo12 | 100 (100) | FJ515676 |
| Rpo13 | 88 (99) | FJ515677 |
