# Supplementary material for: Evolution of Complex RNA Polymerases: The Complete Archaeal RNA Polymerase Structure
Source: PLoS Biol. 2009 May 5;7(5):e1000102. doi: 10.1371/journal.pbio.1000102 (PMC2675907; doi:10.1371/journal.pbio.1000102)
Supplement: Text S1 — (41 KB DOC) [file pbio.1000102.sd004.doc]

**Text S1**

**Model building and Refinement Protocol**

The sequence for each archaeal subunit was determined by PCR mediated cloning and sequencing (see below) and docked onto the poly-alanine model. In this process we used as a guide the structure of the eukaryotic PolII (PDB entry 1WCM) with the corresponding sequence alignments (with exception of Rpb9 that is absent in the archaeal polymerase). Rigid-body, positional and B-overall refinement was repeated with the updated RNAP model in both crystal forms (*P*2_1_2_1_2_1_ and *P*2_1_2_1_2) using REFMAC [S1] and the cross-averaged map re-analysed (GAP; unpublished program D.I.S.). Then the refinement against the data in *P*2_1_2_1_2 space group (Crystal_2) was re-started using the coordinates of the RNAP structure from *Sulfolobus solfataricus* (PDB entry 2PMZ) [S2]. The atomic models of the clamp-head and jaw domain in Rpo1 and of the Rpo8 subunits were manually rebuilt in COOT [S3] guided by the 2Fo-Fc and Fo-Fc sigmaA-weighted maps and using as reference the eukaryotic homologs. The rebuilt structures were then regularized using CALPHA program [S4]. Modelling of Rpo13 (see below for the identification) was initially achieved by fitting in density two antiparallel idealized poly-alanine α-helices in COOT [S3]. At this stage the B factor of each subunit were refined in CNS [S5] and positional and B overall refinement followed in REFMAC [S1]. To decrease residual errors and/or model bias we alternated the positional refinement of the RNAP model against the two dataset (*P*2_1_2_1_2_1_ *vs.* *P*2_1_2_1_2). Also to improve refinement convergence for the Crystal_2 structure (P*2_1_2_1_2* data) we used the normal-mode method [S6] to anisotropically refine the overall thermal parameters whilst for the P*2_1_2_1_2_1_* data the positional refinement was carried out with two-fold NCS restraints, individual TLS and B overall in REFMAC [S1]. Then Rpo13 sequence was assigned, however this required additional restraints provided by secondary structure and disordered prediction programs (ProteinPredict server [S7]; SCRATCH [S8], RONN [S9]). The 2Fo-Fc and Fo-Fc sigmaA-weighted maps together with this information restricted the register of the central sequence (residues ~38-82) onto the poly-alanine model previously fitted (Figure S4). The manual adjustment of the residues was also aided by refining against sharpened Fobs (a B factor = -50 Å^2^ was applied in XPLOR [S10]). Simulated annealing (starting temperature=6000 Kelvin and harmonic restraint constant=30 Kcal mole^-1^ Å^-2^) in CNS program [S5] was also performed against the data at 3.35 Å omitting in turn from the model the newly identified structural elements. The sigmaA-weighted Fo-Fc omit map was in each case calculated and showed positive density in correspondence of the omitted part (data not shown) confirming the presence of the rebuilt subunits and domains.

**Supporting References**

S1 CCP4 (Collaborative Computational Project, Number 4) (1994). The CCP4 suite: programs for protein crystallography. *Acta Crystallogr D* 50: 760-763.

S2 Hirata A, Klein BJ, Murakami KS (2008) The X-ray crystal structure of RNA

polymerase from Archaea. *Nature* 451: 851-854.

S3. Emsley P, Cowtan K (2004) Coot: model-building tools for molecular graphics. *Acta Crystallogr D* 60: 2126-2132.

S4 Esnouf RM (1997) Polyalanine reconstruction from Calpha positions using the program CALPHA can aid initial phasing of data by molecular replacement procedures. *Acta Crystallogr D* 53: 665-672.

S5 Brunger AT, *et al.* (1998) Crystallography & NMR system: A new software suite for macromolecular structure determination. *Acta Crystallogr D* 54: 905-921.

S6 Poon BK, *et al.* (2007) Normal mode refinement of anisotropic thermal

parameters for a supramolecular complex at 3.42-Å crystallographic resolution. *Proc Natl Acad Sci USA* 104: 7869-7874.

S7 Rost B, Yachdav G, Liu J (2004) The PredictProtein server. *Nucleic Acids Res* 32: W321-326.

S8 Cheng J, Randall AZ, Sweredoski MJ, Baldi P. (2005) SCRATCH: a protein structure and structural feature prediction server. *Nucleic Acids Res* 33: W72-76.

S9 Yang ZR, Thomson R, McNeil P, Esnouf RM (2005) RONN: the bio-basis function neural network technique applied to the detection of natively disordered regions in proteins. *Bioinformatics* 21: 3369-3376.

S10 Brunger AT (1992) X-PLOR, Version 3.1: A system for X-ray Crystallography and NMR (New Haven, CT: Yale University Press).
