# Supplementary material for: Evolution of Complex RNA Polymerases: The Complete Archaeal RNA Polymerase Structure
Source: PLoS Biol. 2009 May 5;7(5):e1000102. doi: 10.1371/journal.pbio.1000102 (PMC2675907; doi:10.1371/journal.pbio.1000102)
Supplement: Text S2 — (32 KB DOC) [file pbio.1000102.sd005.doc]

**Protocol S2**

**Modelling of the Pre-Initiation-Complex**

The model of the archaeal pre-initiation-complex was obtained by assembling on our archaeal RNAP platform the archaeal TBP/TFB/promoter complex (PDB entry 1D3U) [S11]. This superposition used the helical density region docking site corresponding to the first α helix of the TFIIB C-terminal domain in the eukaryotic Pol II-TFIIB complex (PDB entry 1R5U) [S12]. The structural matching was carried out with the Structural Homology Program (SHP, [S13]) and subunit Rpo1 was used as reference for the superposition of equivalent eukaryotic subunits. Similarly, to gather information on the spatial organization between our archaeal RNAP and the DNA-RNA hybrid during elongation, we docked the Rpb1-DNA-RNA Pol II elongation complex (PDB entry 1R9T) [S14] onto Rpo1 (2.8 Å rmsd, 1153 Ca equivalences).
