# Supplementary material for: Correction: Evolution of Complex RNA Polymerases: The Complete Archaeal RNA Polymerase Structure
Source: PLoS Biol. 2010 Aug 20;8(8):10.1371/annotation/042f2803-a625-4b2a-bd08-ce50799e4cf6. doi: 10.1371/annotation/042f2803-a625-4b2a-bd08-ce50799e4cf6 (PMC2927221; doi:10.1371/annotation/042f2803-a625-4b2a-bd08-ce50799e4cf6)
Supplement: Supplementary file 1 [file pbio.042f2803-a625-4b2a-bd08-ce50799e4cf6.s001.doc]

**Text S2**

**Modelling of the Pre-Initiation-Complex**

The model of the archaeal pre-initiation-complex was obtained by assembling on our archaeal RNAP platform the archaeal TBP/TFB/promoter complex (PDB entry 1D3U) [S11]. This superposition used the helical density region docking site corresponding to the first α helix of the TFIIB C-terminal domain in the eukaryotic Pol II-TFIIB complex (PDB entry 1R5U) [S12]. The structural matching was carried out with the Structural Homology Program (SHP, [S13]) and subunit Rpo1 was used as reference for the superposition of equivalent eukaryotic subunits. Similarly, to gather information on the spatial organization between our archaeal RNAP and the DNA-RNA hybrid during elongation, we docked the Rpb1-DNA-RNA Pol II elongation complex (PDB entry 1R9T) [S14] onto Rpo1 (2.8 Å rmsd, 1153 Ca equivalences).

**Supporting References**

S11 Littlefield O, Korkhin Y, Sigler PB (1999) The structural basis for the oriented assembly of a TBP/TFB/promoter complex. *Proc Natl Acad Sci USA* 96: 13668-13673.

S12 Bushnell DA, Westover KD, Davis RE, Kornberg RD (2004) Structural basis of transcription: an RNA polymerase II-TFIIB cocrystal at 4.5 Angstroms. *Science* 303: 983-988.

S13 Stuart DI, Levine M, Muirhead H, Stammers DK (1979) Crystal structure of cat muscle pyruvate kinase at a resolution of 2.6 Å. *J Mol Biol* 134: 109-142.

S14 Westover KD, Bushnell DA, Kornberg RD (2004) Structural basis of transcription: separation of RNA from DNA by RNA polymerase II. *Science* 303: 1014-1016.
